# Elemental composition, rare earths and minority elements in organic and conventional wines from volcanic areas: The Canary Islands (Spain)

**DOI:** 10.1371/journal.pone.0258739

**Published:** 2021-11-03

**Authors:** Pablo Alonso Gonzalez, Eva Parga-Dans, Paula Arribas Blázquez, Octavio Pérez Luzardo, Manuel Luis Zumbado Peña, María Mercedes Hernández González, Ángel Rodríguez-Hernández, Carmelo Andújar

**Affiliations:** 1 Department of Agrobiotechnology, IPNA-CSIC, Canary Islands, Spain; 2 Department of Clinical Sciences—Research Institute of Biomedical and Health Sciences, ULPGC, Canary Islands, Spain; 3 Spanish Biomedical Research Center in Physiopathology of Obesity and Nutrition (CIBERObn), Madrid, Spain; De Montfort University, UNITED KINGDOM

## Abstract

The organic wine market is rapidly growing worldwide, both in terms of production and consumption. However, the scientific literature is not conclusive regarding differences in the elemental composition of wines according to their production method, including both major and trace elements. Minerals can be present in wine as a result of both anthropogenic and environmental factors. To date, this has not been evaluated in volcanic contexts, neither has the emergent issue of rare earths and other minority elements as potential sources of food contamination. This study using inductively coupled plasma mass spectrometry (ICP-MS) analyses organic and conventional wines produced in the Canary Islands (Spain), an archipelago of volcanic origin, to compare their content of 49 elements, including rare earths and minority elements. Our results showed that organic wines presented lower potential toxic element content on average than their conventional counterparts, but differences were not significant. Geographical origin of the wine samples (island) was the only significant variable differentiating wine samples by their composition profiles. By comparing our data with the literature, no agreement was found in terms of differences between organic and conventionally-produced wines. This confirms that other factors prevail over elemental composition when considering differences between wine production methods. Regarding the toxicological profile of the wines, five samples (three organic and two conventional) exceeded the maximum limits established by international legislation. This highlights the need for stricter analytical monitoring in the Canary Islands, with a particular focus on Cu and Ni concentration, and potentially in other volcanic areas.

## Introduction

Wine, defined as the product resulting from the fermentation of grape must, is one of the oldest and most globally consumed alcoholic beverages. Interest in the analysis of wine’s elemental composition has increased exponentially due to the need to determine its geographical origin and quality, and beyond the latter its toxicological profile. As a food product, wine can contain elements that can contribute to human nutrition or be toxic and deleterious, often depending on their concentration. For instance Cu and Zn, are essential human minerals but can become toxic in high amounts, while other elements like As, Hg, Cd and Pb are ecotoxic and potentially detrimental for humans even at very low doses [1]. Metal or metalloid contamination of foods is a key concern both in Europe [2] and internationally [3]. It is widely accepted that accumulation of heavy metals in the food chain has deleterious consequences for human health [4]. Moreover, the presence of particular metals in wine, even in low quantities, may significantly affect its quality and organoleptic characteristics such as aroma, taste and colour, potentially leading to a range of oenological problems [5].

Metals (for convenience often including the toxic non-metals or metalloids like As and B) enter the wine at various production stages and mechanisms, both natural (primary) and anthropogenic (secondary) [6]. The first and most commonly reported source is the vineyard’s environment -including air pollution- and soil properties, which also depend on the vines’ absorbance capacities [7, 8]. Both natural and artificial soil components can enter grapes and consequently the wine. The eventual presence of potentially toxic elements in wines depends on the geological origin and type of soil and on the employment of fertilisers and pesticides, both carrying an array of heavy metals [9, 10]. Fertilisers present high concentrations of Cd, Co, Cu, Zn, Mn and Pb, especially the superphosphates employed in conventional agriculture [11]. In turn, toxic elements contained in pesticides can end up in wines both through residual presence in soils and direct application to vines, especially As, Co, Cr, Ni and Pb [12].

The second source of contamination comes from the dozens of oenological additives permitted by the varying national legislations, for use during the initial winemaking phases such as alcoholic fermentation [13]. Fining agents such as bentonite or copper sulphate are well-known carriers of various metals [14]. Finally, such contaminations can occur during subsequent wine processing, contact with winery equipment, and wine storage and bottling conditions, including leaching from glass and cork [13]. A recent in-depth review of elements contained in wine has shown the complexities involved in ascertaining their origin in each specific wine sample [15, 16]. The potential deleterious effects of cumulative risk element ingestion through wine consumption in human health led the OIV (*Organisation Internationale de la Vigne et du Vin*) to introduce standard maximum acceptable limits of As, B, Br, Cd, Cu, Pb and Zn concentrations [4]. Other countries such as Croatia or Germany have established more stringent regulations, imposing concentration limits for Ag, Al, B, Cr, F, Na, Ni, Sb and Sn for instance [5].

The same limits apply to both organic and conventional wines, although it was initially thought that the former would present a lower amount of toxic metals due to the prohibition of synthetic fertilisers and plant protection products under organic standards [17]. However, recent reviews reach no agreement on the matter, and the few available comparative studies do not show significant differences in trace element content between organic and conventional wines [18]. Most studies comparing organic and conventional are focused on chemical composition, productivity, antioxidant compounds (anthocyanins, polyphenols, resveratrol, flavonoids etc.) and pesticide residues [19]. However, given the importance of the burden of potential toxic elements in wine safety and quality, this study aims to advance knowledge about the composition of organic and conventional wines by analysing a hitherto under-researched setting such as the Canary Islands (Spain). Moreover, the continuous growth in surface area of organic vineyards, and their production and consumption worldwide makes this topic timely and relevant [20].

Owing to environmental and geological variations between the seven islands, the present study also aims to explore whether production method or geographical origin has more impact on wine’s elemental profile. A number of studies have explored the general mineral content of wines from the archipelago [21–23], estimated their toxic potential [24], and attempted to identify wine provenance and characteristics according to island [25, 26]. However, no study has yet considered the comparison between organic and conventional production methods, despite the Canaries being the most pesticide-intensive region in Spain [27, 28]. Knowledge about metal content in wines from volcanic settings is also lacking [29]. Likewise, as Gajek, Pawlaczyk (15) point out, most earlier studies are limited to a few elements. Instead, our study provides data on 49 elements including the rare earth elements (REE) and other minority elements (ME), whose significance in wines and the food chain is only starting to be considered [30–32].

The Canary Islands are a Spanish archipelago of volcanic origin comprising seven main islands, located almost 100 km west of the African shore. All the islands have significant winemaking traditions both commercially and for self-consumption, currently comprising ten designations of origin recognised by the European Union. The island of Tenerife has most vineyard surface and production with five designations of origin. The total harvest in 2020 reached 7,655,715 kg [33], while the total vineyard surface area was 8076 ha in 2019 [34]. Of those, 414 ha or 5.1% were certified organic, with 27 registered organic cellars [35]. To date, no studies have addressed topics commonly found in the comparative literature about organic and conventional agriculture in the archipelago, such as productivity, pesticide residues, product quality, chemical composition and elemental profile. This study examines 49 elements for potential use in distinguishing Canary organic wines from their conventional counterparts, on each wine-producing island. ICP-MS was employed to examine the elemental composition of wines and classify them through statistical analysis.

## Material and methods

### Wine samples

A total of 14 wines, two per wine-producing island were chosen during the vintage 2019–2020. Detailed information about each wine is provided in Table 1. Wine names were coded according to the island of origin and production method. Their original names and geolocation remains hidden for privacy purposes in this paper. The sampling strategy consisted of choosing pairs of organic and conventional wines produced from vineyards as close as possible to each other, mostly in a radius of a few km, to minimise differences in soil and climate characteristics. Different winemaking methods were also taken into account and a total of six red and eight white wines were selected. The sampling also prioritised pairs of wines with similar profiles in terms of alcohol volume, residual sugar, harvest year, grape variety and ageing tanks employed. Most wines were dry, except the conventional sample from El Hierro, a sweet wine analysed owing to the lack of alternative wine samples in the area. All wines came from protected designations of origin except the organic wine samples from Tenerife and Fuerteventura. All samples were collected personally in the cellars, from commercially available bottles rather than wines stored in cellars until commercialisation. All organic wines were certified by the Canary Institute of Agrofood Quality (ICCA) under the EU organic agriculture scheme. The organic samples from Tenerife and La Gomera were in their second year of the compulsory transition period to organic agriculture when samples were collected. All samples were transferred to plastic containers after the original bottles were opened, and stored at 4-5°C until analysis.

**Table 1:** Sample description including codification, island, type of wine, production method, harvest, grape variety, location, and geological substrate.

Sample	Island	Type	Production	Harvest	Variety	Site	Geological Substrate
TF1	Tenerife	Red	Organic & Biodynamic	2019	Listán Negro	La Perdoma	Basaltic lava flows
TF2	Tenerife	Red	Conventional	2019	Listán Negro	La Perdoma	Basaltic lava flows
LP1	La Palma	White	Organic	2019	Albillo Criollo	Puntagorda	Basaltic lava flows
LP2	La Palma	White	Conventional	2019	Albillo Criollo, Listán Blanco	Tijarafe	Basaltic lava flows
GC1	Gran Canaria	Red	Organic	2019	Listán Negro, Castellana	Vega de Gáldar	Basanitic-nephelineitic, basaltic and olivine-pyroxenic basaltic lavas
GC2	Gran Canaria	Red	Conventional	2019	Listán Negro	Vega de Gáldar	Basanitic-nephelineitic, basaltic and olivine-pyroxenic basaltic lavas
LG1	La Gomera	White	Organic	2019	Forastera Gomera	Igualero	Basaltic and trachybasaltic lava flows
LG2	La Gomera	White	Conventional	2019	Forastera Gomera	El Cercado	Basaltic and trachybasaltic lava flows
FT1	Fuerteventura	White	Organic	2019	Marmajuelo, Malvasía	Casillas de Morales	Colluvium and slope deposits
FT2	Fuerteventura	White	Conventional	2019	Malvasía	Lajares	Basaltic lava flows
EH1	El Hierro	White	Organic	2019	Verijadiego, Pedro Ximenez, Listán Blanco	Frontera	Basaltic, basanitic and tephritic lava flows
EH2	El Hierro	White	Conventional	2019	Verijadiego	Frontera	Basaltic, basanitic and tephritic lava flows
LZ1	Lanzarote	Red	Organic	2019	Listán Negro, Syrah	La Geria	Dispersion pyroclasts
LZ2	Lanzarote	Red	Conventional	2019	Listán Negro, Syrah, Tintilla, Merlot	La Geria	Dispersion pyroclasts

Geological data retrieved online from the “Continuous Digital Geological Map of the Canary Islands”, last updated in 2010. See https://www.idecanarias.es/listado_servicios/mapa-geologico.

### Elements and standard curves

A total of 49 elements were analysed (see S1 Table), including essential nutrients, those included in the priority list of the Agency for Toxic Substances and Disease Registry (ATSDR), and also rare earth (REEs) and other minority elements (MEs). Thus, a very broad spectrum was covered in this study. These ranged from essential dietary elements to those considered emerging pollutants, due to their increasing environmental release through massive use in the manufacture of electrical and electronic devices [36], and including heavy metals and metalloids classically considered toxic.

Pure element standards, purchased in 5% HNO3 solution (CPA Chem, Stara Zagora, Bulgaria), were used to prepare calibration lines. To avoid polyatomic interference, two complementary calibration lines were prepared as described in previous work [37]. One contained the essential elements and those from the ATSDR list, and the other curve contained the REE and ME. Both straight lines covered the range 0.005–300 ng/ml (ng/ml), prepared at 10 levels.

### Analytical procedure

Wine samples were first subjected to vigorous agitation by Vortex. After that, they were placed in an ultrasonic equipment for 30 minutes, in order to dissolve possible aggregates by sonication. Once sonicated, 1 ml of each was deposited in a digestion vessel and agitated with 1 ml of 65% concentrated nitric acid. The acid had been previously ultrapurified by distillation in our laboratory until the detectable concentrations of each element fell below its limit of quantification (LOQ). Eight ml of ultrapure water (MilliQ) was then added. Once the digestion vessels were closed, the samples were digested in a Milestone Ethos Up microwave oven, using the following schedule: Step 1: power (W), temperature (C), and time (min) of 1800, 100, and 5, respectively; Step 2: 1800, 150, and 5; Step 3: 1800, 200, and 8; Step 4: 1800, 200, and 7, as previously described (Rodríguez-Hernández et al., 2019). The digested sample was then transferred to a 15 ml polypropylene tube without further dilution (final concentration 4%). Four 2 ml aliquots were transferred from each tube into an autosampler vial for subsequent determination of the elements by ICP-MS. A reagent blank, prepared as for the samples, was included every 14 samples in the analytical batch.

For this quantitative element analysis, an Agilent 7900 ICP-MS equipped with Agilent MassHunter software (version 4.2, Agilent Technologies, Palo Alto, CA, USA) was used for data acquisition and processing. The entire procedure was earlier employed with different types of food samples in our laboratory. When applied to wine samples, the entire procedure was validated for this matrix prior to use, using in-house fortified samples. All determinations were performed in triplicate from each vial. Thus, for each wine sample, 12 measurements were obtained. The recoveries obtained ranged from 83 to 119% for toxic and essential elements. Linear calibration curves were found for all elements (regression coefficients ≥ 0.997). Limits of detection (LOD) and quantification (LOQ) were calculated as the concentrations that respectively produced signals three and ten times higher than the averaged blanks. Sample LOQ were calculated by multiplying the instrumental LOQ by the dilution factor (1:10 v:v). LODs are presented in S2 Table.

### Statistical analysis

Correlations between all pairs of the 49 element concentration results obtained were tested using Spearman correlation coefficients. We then tested for significant differences (Wilcoxon rank-sum and signed-rank tests using ‘island’ as the pairing factor) between each element concentration measured in wine samples from conventional and organic production methods. We also applied a principal component analysis (PCA) of the standardised measurements to establish a multivariate ordination of the wine samples according to their elemental composition, and to visualise its correspondence with the origin of these wine samples according to island (El Hierro, La Palma, La Gomera, Tenerife, Gran Canaria, Fuerteventura or Lanzarote), type of wine (red or white), and production method (conventional or organic). Finally, to assess significant differences between groups of wine samples according to their elemental concentration profiles, permutational ANOVAs were conducted over a Euclidean distance ordination matrix based on the standardised measurements. This used 999 permutations and the geographical origin, wine type or production method as grouping factors. The data analyses and plots were carried out using the R-packages corrgram, corrplot, vegan and ade4 by R Development Core Team.

## Results and discussion

### Elemental compositional profiles and their major drivers in wines from the Canary Islands

**Fig 1:**
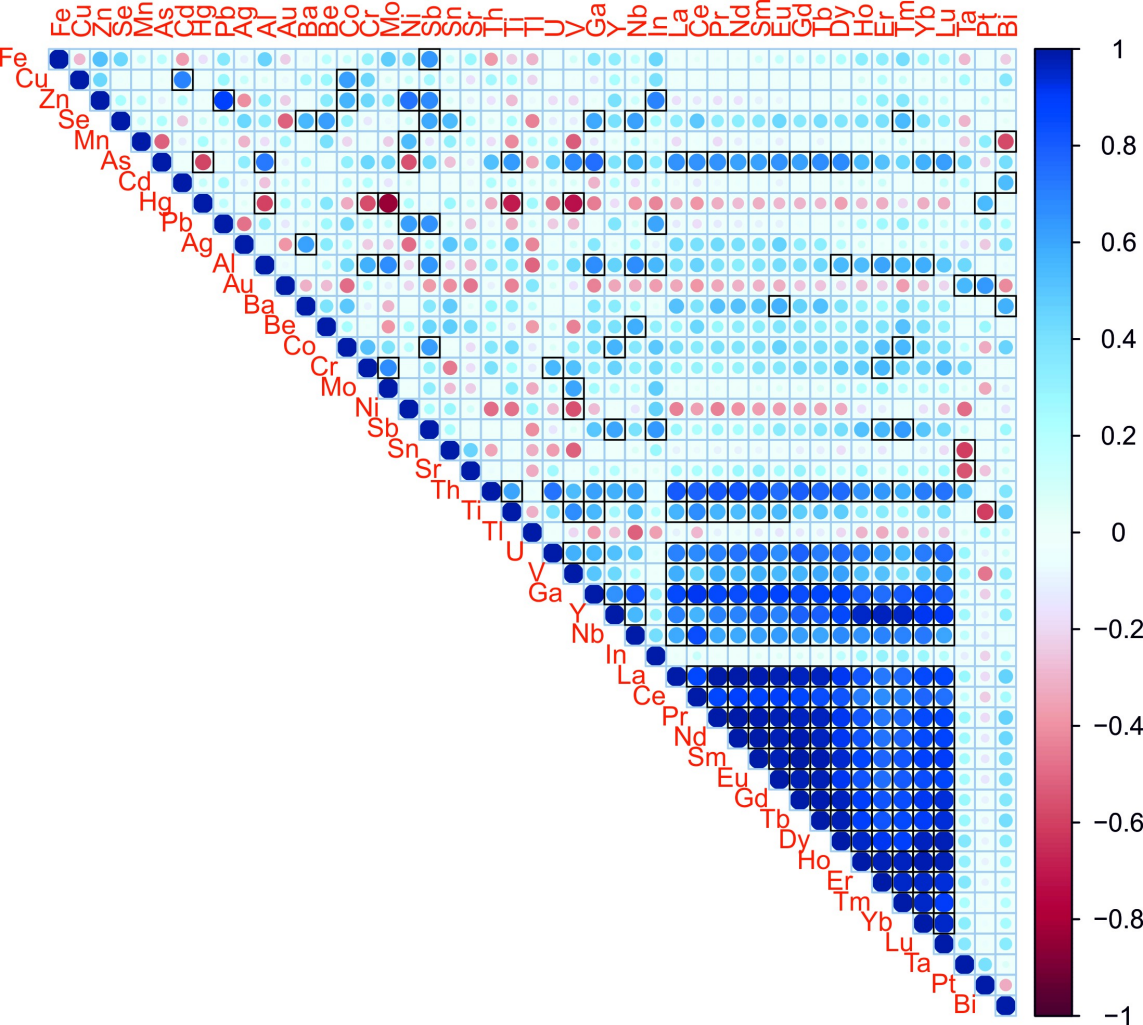
Correlation plot between all pairs of the 47 metal concentration measures obtained. Color scale according to Spearman correlation coefficient (r) and circle size absolute r values. Significant differences (p < 0.05) are black framed.

**Fig 2:**
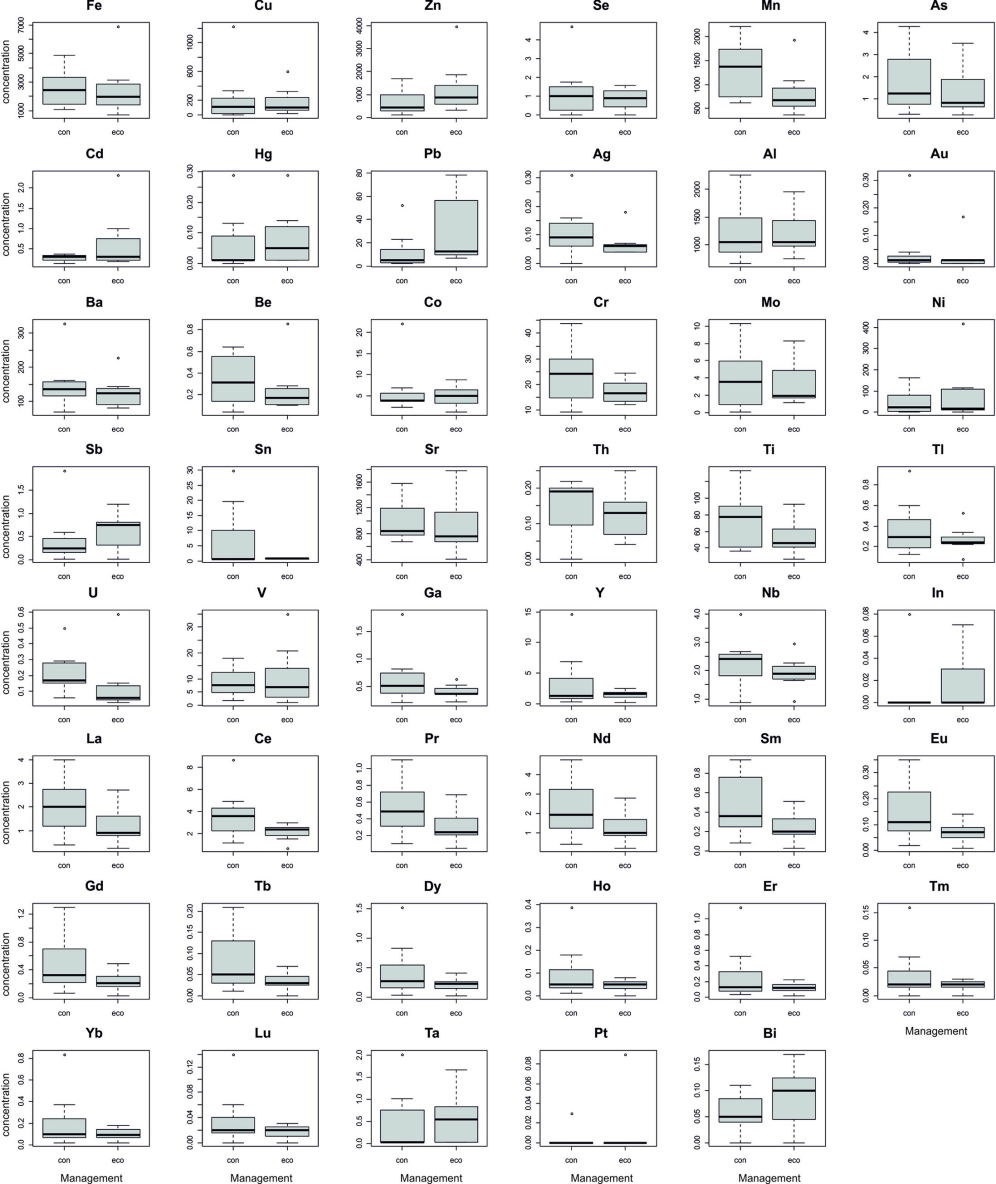
Metal concentrations for wine samples from conventional management (con) and organic management (eco). See S1 Table for concentration units of each variable.

The results of the analysis of 49 elements in the 14 samples are summarised in S1 Table, where the mean and standard deviations of the twelve measurements (technical replicates) are shown for each wine sample. The mean was preferred over the median as a central indicator for comparisons as is common in the literature [15]. An overall positive correlation across the 49 elemental concentrations was found (Fig 1). Significant and strong positive correlations (r > 0.75) were found between rare earth elements and for As, Th, Ti, U, V, Ga and Nb. Hg was negatively correlated with other elements, significantly and strongly (r < -0.75) with Al, Cr, Mo, Ti and V (Fig 1). Wine samples from conventional production tended to have a higher mean concentration for most of the measured elements (with the exception of Zn, Cd, Hg, Pb or Bi), and heterogeneity in the measurements also tended to be higher than in the wine samples from organic management (Fig 2). However, significant differences in concentrations (Wilcoxon rank-sum and signed-rank tests, p < 0.05) between wine samples from conventional and organic management were not found for any of the assayed elements.

**Fig 3:**
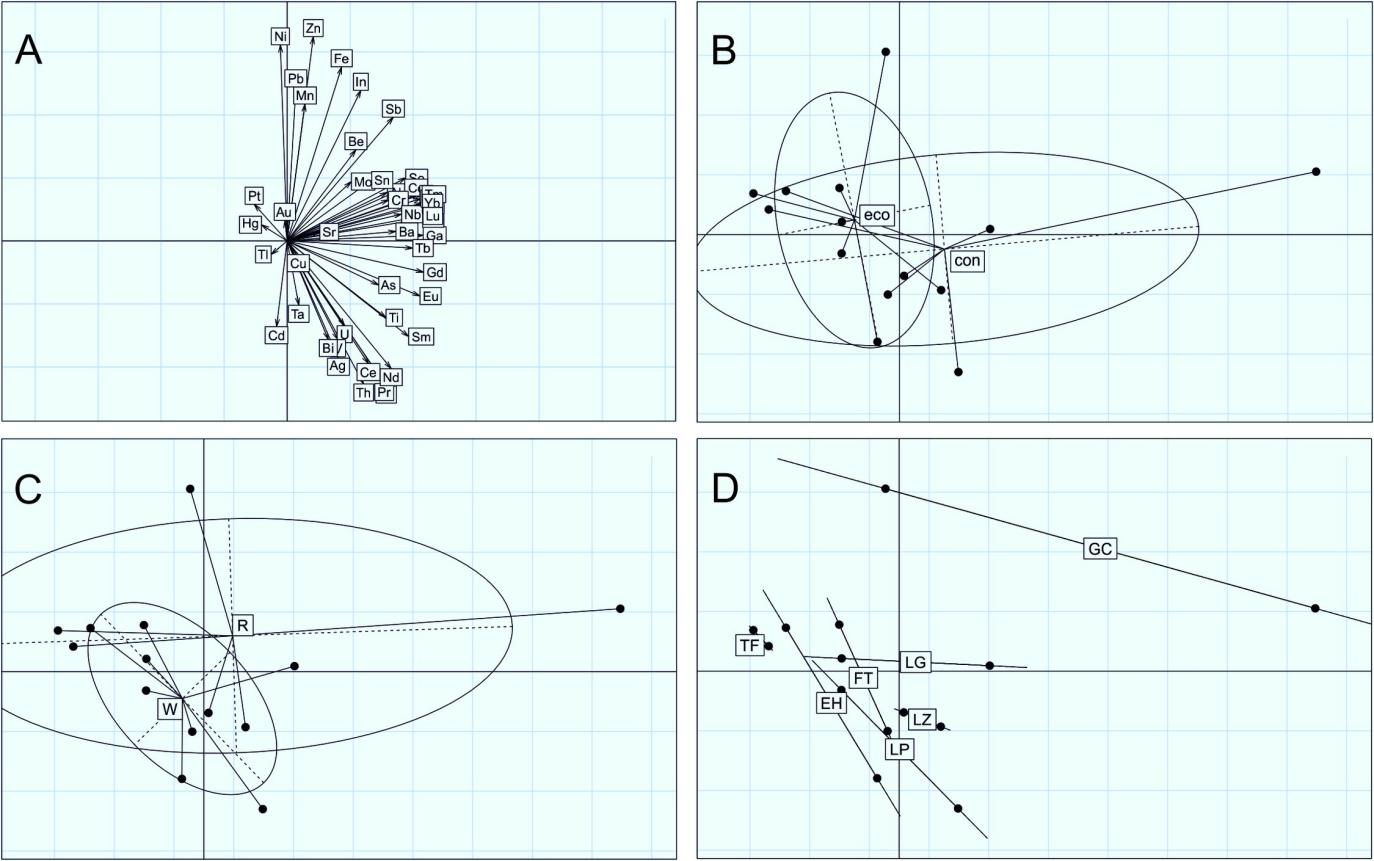
A-D. Principal components analyses ordinations of the wine samples according to the variation in metal concentration profiles. A) variable contribution to two main principal components and B, C, D) wine samples ordinations grouped by management type (conventional, con; organic, eco), type of wine (red, R; white, W) and the island of origin (El Hierro, EH; La Palma, LP; La Gomera, LG; Tenerife, TF; Gran Canaria, GC; Fuerteventura, FT; Lanzarote, LZ) respectively.

The first two axes of the PCA of elemental composition profiles for the wine samples accounted for 57% of total variance. PC1 was positively and strongly correlated with Ga, Y, Eu, Gd, Dy, Ho, Er, Tm, Yb, and Lu; and PC2 was positively correlated with Zn, Fe, Ni and negatively with Cd (Fig 3A). In the ordination scatter plots using pairs PC1 and PC2, no clear ordination of samples according to production method or wine type was observed (Fig 3B and 3C). Considering their island of origin, the ordinations showed differentiation of wine samples from Gran Canaria (with a high heterogeneity between the two GC wine samples) and a secondary ordination across the PC1 in agreement with the other islands (Fig 3D). Accordingly, permutational ANOVAs revealed non-significant differences in the elemental composition profiles of the wine samples between production methods (p = 0.365, r^2^ = 0. 081) and wine types (p = 0.507, r^2^ = 0. 070). However, significant differences among islands were found (p = 0.047, r^2^ = 0. 549), and this result was maintained in the subset of wine samples on excluding Gran Canaria wines (p = 0.027, r^2^ = 0.557).

Despite some general trends for conventional wines showing higher (but non-significant) concentrations for many individual elements, sample compositions did not significantly differentiate wines from the two types of management procedures (Table 2). This result converges with previous research analysing major and trace elements in organic and conventional wines, showing no clear differential profiles between them [19, 38–40]. In our study, possible differences in element composition between wine management types may have remained undetected because a higher level of replication was needed, particularly considering the heterogeneity found across the wines of the different islands (see discussion below). Otherwise, it is clear that there was no important variation in elemental composition profiles between conventional and organic wines in our dataset, even when the island was considered as a pairing factor (Wilcoxon signed-rank test). Still, looking at individual concentrations, conventional wine samples tend to presented higher (but non-significant) levels of Cu, Se, Mn, Ag, Al, Au, Ba, Be, Co, Cr, Mo, Sn, Sr, Th, Ti, Tl, U, Ga, Y, Nb, La, Ce, Pr, Nd, Sm, Eu, Gd, Tb, Dy, Ho, Er, Tm, Yb, and Lu, while organic wines had higher concentrations of Fe, Zn, Cd, Hg, Pb, Ni, Sb, V, In, Ta, Pt and Bi. Čepo et al. [19] found that organic wines presented lower Pb levels, while in our study the contrary applies. The results of Vrček et al. [41] only converge with our study in the higher concentrations of Ni and Vi in organic, and of Se, Cr and Mo in conventional wines.

**Table 2:** Concentration of each element in all organic and conventional samples.

Element	Total Organic	Total Conventional
**56 Fe**	2,602.30	2,574.94
**63 Cu**	186.27	264.41
**66 Zn**	1,304.51	689.19
**78 Se**	0.83	1.31
**55 Mn**	848.6	1,308.55
**75 As**	1.37	1.83
**111 Cd**	0.68	0.28
**202 Hg**	0.09	0.07
**208 Pb**	32.99	13.74
**107 Ag**	0.07	0.11
**27 Al**	1,225.59	1,241.52
**197 Au**	0.03	0.06
**137 Ba**	127.07	154.69
**9 Be**	0.27	0.34
**59 Co**	4.9	6.7
**52 Cr**	17.29	23.79
**95 Mo**	3.48	3.97
**60 Ni**	96.03	49.73
**121 Sb**	0.6	0.49
**118 Sn**	0.71	7.54
**88 Sr**	935.13	1,004.71
**232 Th**	0.12	0.14
**47 Ti**	53.26	72.77
**205 Tl**	0.27	0.38
**238 U**	0.15	0.23
**51 V**	11.06	8.8
**71 Ga**	0.41	0.68
**89 Y**	1.45	3.75
**93 Nb**	1.92	2.3
**101 Ru**	0	0
**115 In**	0.02	0.01
**139 La**	1.23	2.05
**140 Ce**	2.12	3.8
**141 Pr**	0.32	0.54
**146 Nd**	1.29	2.28
**147 Sm**	0.25	0.49
**153 Eu**	0.07	0.15
**157 Gd**	0.24	0.5
**159 Tb**	0.04	0.08
**163 Dy**	0.21	0.47
**165 Ho**	0.04	0.11
**166 Er**	0.12	0.3
**169 Tm**	0.02	0.04
**172 Yb**	0.1	0.22
**175 Lu**	0.02	0.04
**181 Ta**	0.57	0.52
**189 Os**	0	0
**195 Pt**	0.01	0
**209 Bi**	0.09	0.06

Results are provided as means in ng/l.

The main result found by Korenovská and Suha [42] was that organic wines had lower levels of Fe and Cu, and that both metals have the potential to be employed in distinguishing between the two production methods. However, in our study, differences were non-significant, and in any case organic wines presented lower Cu but higher Fe levels. Another notable result is that conventional wines tend to present higher (but non-significant) concentrations of *rare earths* than organic wines. No previous research has focussed specifically on whether the organic production method influences rare earth composition against conventional methods, although some studies point to associated geographical wine profiles [43]. The most plausible explanation for the higher levels of rare earths in conventional wines derives from the use of more processing additives, e.g. bentonite and diatomaceous earth and physical treatments, e.g. filtration and storage [44].

Our results only showed significant differences in the elemental composition of wine samples according to their geographical (island) origin (see Table 3 for average concentrations per island). Gran Canaria wines presented the overall highest amount of the most important elements among all the Canary Islands, including Fe, Zn, Se, Mn, Al, Ba, Be, Co, Cr, Mo, Ni, Sb, Sn, Ti, Y, Nb, In, Eu, Gd, Tb, Dy, Ho, Er, Tm, Yb and Lu. In the case of some key elements such as Fe, Zn and Al, wines from Gran Canaria almost doubled the average of any other island. This could be explained by the location of both vineyards near a high-transit highway and in soils previously occupied by banana plantations, which have been usually subjected to intensive application of pesticides and fertilisers. Another remarkable result of the analysis is that the mean concentration of Ni for Gran Canaria and La Gomera) exceeded the OIV limit of 100 ng/ml, respectively reaching 238.68 and 106.05 ng/ml. Regarding the rest of the islands, wine samples tended to group together by island, but the heterogeneity among islands was high, particularly for La Gomera, Fuerteventura, La Palma and El Hierro.

**Table 3:** Concentration of each element in ng/l, averaging the data for the two samples analysed per island.

Element	TF	LP	GC	LG	FT	EH	LZ
**56 Fe**	1,712.23	2,050.22	5,908.13	1,909.45	1,288.99	2,149.13	3,102.16
**63 Cu**	54.48	165.92	212.16	149.62	914.42	58.40	22.40
**66 Zn**	474.73	496.08	2,830.03	1,085.56	1,146.57	546.09	398.88
**78 Se**	0.56	1.21	3.14	1.19	0.11	0.00	1.28
**55 Mn**	1,369.17	547.75	1,653.15	1,642.72	639.16	1,095.10	602.97
**75 As**	0.40	1.18	2.41	0.70	1.79	0.83	3.89
**111 Cd**	0.28	0.57	0.28	0.34	0.41	1.27	0.21
**202 Hg**	0.13	0.17	0.02	0.19	0.01	0.01	0.01
**208 Pb**	8.10	7.56	46.10	47.68	40.45	6.79	6.87
**107 Ag**	0.07	0.24	0.10	0.05	0.08	0.02	0.08
**27 Al**	864.66	859.96	1,943.91	990.08	1,194.09	906.76	1,875.42
**197 Au**	0.10	0.00	0.00	0.16	0.01	0.01	0.01
**137 Ba**	116.70	153.07	204.84	190.03	115.23	76.40	129.90
**9 Be**	0.19	0.42	0.71	0.41	0.21	0.07	0.11
**59 Co**	1.76	4.35	13.07	6.58	7.31	3.04	4.51
**52 Cr**	12.00	11.47	30.20	19.89	28.33	20.62	21.26
**95 Mo**	1.32	0.60	6.40	1.25	5.46	4.98	6.08
**60 Ni**	19.59	10.67	238.68	106.05	51.88	81.47	1.81
**121 Sb**	0.22	0.49	1.56	0.46	0.47	0.02	0.61
**118 Sn**	10.18	0.76	15.30	0.71	0.65	0.65	0.65
**88 Sr**	789.22	1,674.90	1,341.72	690.40	600.99	975.78	716.45
**232 Th**	0.02	0.13	0.12	0.18	0.17	0.14	0.16
**47 Ti**	34.03	59.99	86.10	54.11	68.98	66.86	71.05
**205 Tl**	0.26	0.27	0.10	0.74	0.25	0.42	0.23
**238 U**	0.04	0.11	0.21	0.27	0.16	0.38	0.15
**51 V**	1.34	6.23	9.35	4.13	7.74	14.24	26.47
**71 Ga**	0.22	0.59	1.17	0.44	0.41	0.36	0.65
**89 Y**	0.26	1.47	8.39	4.15	0.95	1.06	1.92
**93 Nb**	1.46	1.67	3.49	2.19	2.11	1.57	2.28
**101 Ru**	0.00	0.00	0.00	0.00	0.00	0.00	0.00
**115 In**	0.00	0.00	0.07	0.00	0.04	0.00	0.00
**139 La**	0.33	2.49	1.91	1.71	1.38	1.48	2.18
**140 Ce**	0.93	5.43	3.76	2.81	2.55	2.10	3.12
**141 Pr**	0.07	0.70	0.50	0.46	0.34	0.35	0.56
**146 Nd**	0.30	3.05	2.29	1.93	1.33	1.36	2.24
**147 Sm**	0.05	0.58	0.57	0.42	0.25	0.27	0.43
**153 Eu**	0.02	0.16	0.21	0.15	0.07	0.06	0.12
**157 Gd**	0.05	0.44	0.75	0.50	0.22	0.25	0.40
**159 Tb**	0.01	0.05	0.12	0.11	0.03	0.03	0.06
**163 Dy**	0.04	0.25	0.88	0.51	0.17	0.19	0.34
**165 Ho**	0.01	0.05	0.22	0.11	0.03	0.04	0.07
**166 Er**	0.03	0.12	0.66	0.32	0.09	0.10	0.18
**169 Tm**	0.00	0.02	0.09	0.04	0.02	0.02	0.03
**172 Yb**	0.02	0.10	0.49	0.23	0.08	0.09	0.14
**175 Lu**	0.00	0.02	0.08	0.04	0.02	0.02	0.02
**181 Ta**	0.29	0.03	0.03	1.02	0.95	0.41	1.08
**189 Os**	0.00	0.00	0.00	0.00	0.00	0.00	0.00
**195 Pt**	0.05	0.00	0.00	0.02	0.00	0.00	0.00
**209 Bi**	0.02	0.14	0.06	0.08	0.08	0.05	0.07

Finally, our results showed no significant differences according to wine type. Average concentrations per wine type are shown in Table 4. Again, a higher number of replicates may be required to infer subtle differences in the profiles between red and white types, but from our study, it is clear that there are no outstanding differences. These results differ from previous studies that argued that wine type is the main variable that determines the elemental composition of wines [15], probably due to different winemaking techniques [45]. In white wines, Gajek et al. [15] reported higher levels of Ag, Be, Bi, Cd, Co, Li, K and Ti, while Płotka-Wasylka et al. [46] reported Ag, Al, As, Bi, Cu, Sb, Se, Sn, Zr and Zn. In Brazil, Mirlean et al. [47] detected higher concentrations of Cu in red than white wines, coinciding with data from other countries [48, 49], probably related to elimination of fungicide containing grape skins before fermentation. In our study, however, Ag, Cd, and Cu presented higher levels in white than reds, while Al, As, Sb, Se, Sn and Zn were higher in reds. In turn, red wines have been characterised by higher values of Be, Ba, Cr, Cu, Mn, Sr and Zn [15], and Be, Ba, Fe, K and Mn [46]. Our study only found higher concentrations of Ba, Cr, Mn, Zn and Fe, while Cu and Sr were higher in whites. Thus, our results can only confirm the higher general levels of Mn and Ba in reds and Ag in whites, deviating from the general conclusions of Greenough et al. [50].

**Table 4:** Average concentration in ng/ l of each element in the two wine types.

Element	White	Red
**56 Fe**	1,849.45	3,574.17
**63 Cu**	322.09	96.35
**66 Zn**	818.58	1,234.55
**78 Se**	0.63	1.66
**55 Mn**	981.18	1,208.43
**75 As**	1.12	2.24
**111 Cd**	0.65	0.26
**202 Hg**	0.10	0.06
**208 Pb**	25.62	20.35
**107 Ag**	0.10	0.08
**27 Al**	987.72	1,561.33
**197 Au**	0.05	0.04
**137 Ba**	133.68	150.48
**9 Be**	0.28	0.34
**59 Co**	5.32	6.45
**52 Cr**	20.08	21.16
**95 Mo**	3.07	4.60
**60 Ni**	62.52	86.69
**121 Sb**	0.36	0.80
**118 Sn**	0.69	8.71
**88 Sr**	985.52	949.13
**232 Th**	0.16	0.10
**47 Ti**	62.48	63.73
**205 Tl**	0.42	0.20
**238 U**	0.23	0.13
**51 V**	8.09	12.39
**71 Ga**	0.45	0.68
**89 Y**	1.91	3.52
**93 Nb**	1.89	2.41
**101 Ru**	0.00	0.00
**115 In**	0.01	0.02
**139 La**	1.77	1.47
**140 Ce**	3.22	2.60
**141 Pr**	0.46	0.38
**146 Nd**	1.92	1.61
**147 Sm**	0.38	0.35
**153 Eu**	0.11	0.12
**157 Gd**	0.35	0.40
**159 Tb**	0.06	0.06
**163 Dy**	0.28	0.42
**165 Ho**	0.06	0.10
**166 Er**	0.16	0.29
**169 Tm**	0.02	0.04
**172 Yb**	0.12	0.22
**175 Lu**	0.02	0.04
**181 Ta**	0.60	0.46
**189 Os**	0.00	0.00
**195 Pt**	0.00	0.02
**209 Bi**	0.09	0.05

Although data for wine type was not statistically significant, certain metal contents such as Fe, Zn or Al revealed differences coherent with results reported in the literature.

### Element concentrations and their maximum limits in wines from the Canary Islands

The mean value of the whole sample for each element was also calculated to perform simple comparisons with other datasets. In some samples, its presence was below the detection limit. Average contents decreased in the following order: Fe > Al > Zn > Sr > Cu > Ba > Mn > Ni > Ti > Pb > Cr > V > Co > Sn > Mo > Ce > Y > Nb > Nd > La > As > Se > Sb & Ga > Ta > Cd > Pr > Sm & Gd > Dy > Tl > Be > Er > U > Yb > Th > Eu > Ag > Ho & Hg > Bi > Tb > Au > Tm & Lu > In > Os & Pt & Ru. The group with the highest concentrations (above 1,000 ng/ml) was Fe and Al. In the next group, concentrations range between 100 and 1000 ng/ml such as Zn, Sr, Cu, Ba and Mn. About one order of magnitude below these, between 1 and 100 ng/ml, we found Ni, Ti, Pb, Cr, V, Co, Mo, Y, As, Ce, Nb and Nd. The lowest levels (below 1 ng/ml) were mostly those of rare earth elements.

Overall similar results were reported in previous studies internationally [15, 51] and in the Canaries, where Perez-Trujillo [23] reported the following concentrations in descending order: Rb > Sr > Zn > Ti > Cu > Ba > V > Ni > Zr > Co > Sn > Cs > W > Sb > Be > Tl > Te > Re > Pt > Au. However, our dataset differs slightly from the averages reported in the literature for some elements [52]. Fe, Al, Be, Ni and Tl presented higher levels than average, while Cu, Mn, As, Cd, Pb, Sb, Sn and V were lower. The volcanic nature of the Canary soils could explain this discrepancy, although the lack of evidence about volcanic wines does not allow us to confirm this. Research in this field has mostly focussed on soils. In their analysis of heavy metals in EU soils, Tóth, Hermann [53] suggest that higher levels of Pb in the Italian Lazio region may be explained by the volcanic nature of the region. In contrast, our data suggest a lower level of Pb on average. Similarly, Peña-Rodríguez, Pontevedra-Pombal [54] support the idea that soils located near volcanic areas show a notable Hg enrichment. However, Hg did not reach high levels in our case. A single study on the volcanic island of Sicily by La Torre, Rando [29] explored four elements in wines. In line with our results, Pb in Sicilian wines was low on average, while Cu and Zn were similar to our dataset, with Cd presenting slightly higher amounts, which could be due to pesticide and fertiliser use. Results are in no way conclusive regarding elemental composition of wines produced in volcanic areas, so more research is certainly needed.

Toxicity levels of the samples did not exceed the maximum acceptable limits according to OIV and various international legislations, except one conventional sample exceeding maximum Cu limits (FT2), and three organic and one conventional sample exceeding Ni levels (GC1, LG1, FT1 and EH2), with a further conventional sample (LG2) almost reaching the legal limit with 99.05 ng/ml. Although the levels of some elements are variable and higher in some wines/soils than in others, the levels found in no case represent a risk for consumers, not even for those in the 97.5th percentile of consumption. Finally, regarding the toxicological profile of wines, we briefly review our results in the light of the literature, considering only elements with maximum concentration limits established by international legislation. While it is true that there is harmonized legislation across the EU, there is also the freedom for countries to impose additional restrictions in certain circumstances, either based on the particular food consumption habits of their population, or any other differentiating fact that they consider could affect food safety. Given that the European Union only establishes maximum limits for lead, cadmium, mercury and inorganic tin, legislations from specific countries whose legislation contemplate limits for other elements will be employed instead.

#### Cu

The presence of Cu in wines is often derived from the use of pesticides against mildew type moulds in the vineyard [55] but can also be related to the use of copper or bronze apparatuses during winemaking to suppress defects such as reduction and hydrogen sulphide [56]. Indeed, Cu is among the oldest plant protection substances employed in vineyards in large amounts, mainly against powdery and downy mildew, and permitted in organic agriculture until recently [57, 58]. The typical example is the widely employed Bordeaux formulation, mixing copper sulphate with calcium hydroxide [59]. In our study, Cu levels were 242.67 ng/ml on average. This is lower than most data reported in the literature, e.g. more than 1,000 ng/ml were found in Brazil [47], Croatia [60], Jordan [61], Serbia [62], Czech Republic [63] and Australia [64].

Although differences are not statistically significant for any element, conventional wines presented slightly higher Cu levels than organic wines on average (264.41 vs 186.27 ng/ml). This is in line with previous studies comparing conventional and organic wines such as Čepo et al. [19], who reported average Cu concentrations of 180 ng/ml and 166.3 ng/ml respectively. However, this contradicts previous studies such as Seralini et al. [65], who argue that organic wines tend to have lower Cu contents than their conventional counterparts in France with an average of 150 ng/ml, lower than reported in our study. The mean of the two wines from Fuerteventura showed the highest concentration of Cu (914.42 ng/ml), while the lowest was found on Lanzarote with 22.40 ng/ml. A sample of conventional wine from Fuerteventura (FT2) exceeded the OIV limit with 1,228.25 ng/ml. The prevalence of fungal diseases in vineyards on Fuerteventura, attributable to their locations close to the sea, low altitudes and high relative humidity, could explain their higher Cu levels than other islands. However, the fact that the organic sample from Fuerteventura (FT1) showed half the Cu concentration of its conventional counterpart could mean these increased Cu levels are related to wine storage and processing in the cellar.

#### Ni

A relatively high number of samples presented Ni concentrations exceeding the maximum limit of 100 ng/ml established by the Croatian national legislation (used as a reference given the lack of maximum limits established for Ni in most international and national legislations), including the organic wine samples GC1 (419.63 ng/ml), LG1 (113.04 ng/ml), FT1 (101.95 ng/ml) and the conventional wine EH2 (161.13 ng/ml). The conventional sample LG2 almost reached the limit (99.05 ng/ml). This could be explained by the volcanic character of their soils. However, other islands with similar soils such as La Palma (10.67 ng/ml) or with markedly volcanic soils such as Lanzarote (1.81 ng/ml) present rather lower Ni levels. Moreover, while GC1 presents high Ni levels, the GC2 sample collected nearby in a similar soil shows low levels (57.73 ng/ml). Previous studies in the Canaries have shown lower concentrations on La Gomera (60 ng/ml), Tenerife and La Palma (about 30 ng/ml), although the eastern islands of Gran Canaria, Fuerteventura and Lanzarote were not studied [24]. Similar Ni concentrations have been found in non-volcanic areas, with levels up to 500 ng/ml in Greek and 200 ng/ml in Jordanian wines [66]. Previous studies showed that high Ni presence reflects contamination with pesticides (especially herbicides) and fertilisers (especially copper sulphate and iron sulphate), and soil origin [12], with higher Ni in soils from arid and semi-arid regions [67]. However, the extent to which Ni transfers from soil to wine is rather unclear in the literature. The most plausible explanation is that Ni contamination derives from fermenting and ageing in stainless steel tanks containing Ni [68]. However, this is contradicted by findings showing no change in Ni content with more storage time in stainless steel and different values independent of wine vessel employed [69]. Organic wines presented notably higher levels of Ni on average than conventional wines (96.02 vs 49.73 ng/ml). A similar result was obtained in Italian white wines by Drava and Minganti [38]. However, wine type did not affect Ni levels significantly. More research is needed in volcanic settings to shed light on how soils influence wine elemental composition. Regarding food safety, Ni has been shown to be a potential allergen at rather low doses [70]. However, a 2020 updated review published by the EFSA shows that the risk of Ni consumption is rather low for the general population [71]. In particular, total diet studies focusing on Ni consumption in the Canary Islands have shown low risk overall [72]. The study considers wine as a source of Ni intake, presenting the higher levels among alcoholic drinks but far from other foodstuffs such as cereals and pulses.

#### Al

None of the samples exceeded the maximum aluminium concentration limit established by the most rigorous Croatian legislation of 10,000 ng/ml. All samples were below 2,000 ng/ml with an average of 1,151.31 ng/ml. This is in line with levels reported in Italy and Spain [73, 74], but higher than Croatian samples [19]. As reported by Čepo et al. [19], organic wines presented slightly lower Al values. The eastern Canary Islands of Gran Canaria, Fuerteventura and Lanzarote presented higher values than the western islands, ranging between 1,000 and 2,000 ng/ml, while none of the western islands exceeded 1,000 ng/ml. This could be related to differences in soil composition, but also to wine processing practices given that bentonite, an additive for wine fining, is an additional Al source [5]. Reds presented higher levels of Al than whites (1,561.33 vs 987.72 ng/ml).

#### As

Arsenic is usually found as a result of high-intensity pesticide and herbicide spraying, winemaking conditions and, secondarily, geological origin [13, 59]. All the samples were far below the limits established by the OIV (200 ng/ml) and the most stringent regulation of Hungary (50 ng/ml), with an average 1.49 ng/ml. This is lower than data reported elsewhere [13, 19, 73]. Organic wines showed slightly lower As levels than conventional ones (1.37 vs 1.83 ng/ml). As with Al, the highest arsenic concentration was found on the eastern islands of Lanzarote (3.89 ng/ml) followed by Gran Canaria (2.41 ng/ml) and Fuerteventura (1.79 ng/ml). Red wines doubled whites in As concentration, which can be explained by the use of diatomaceous earth during the clarification stage [75].

#### Cd

Cadmium contamination can originate from superphosphate fertilisers but also from oenological processes in the cellar [76, 77]. All samples were far below the OIV maximum limit of 10 ng/ml, with an average of 0.47 ng/ml comparable to other European countries [19, 73], but significantly lower than averages found in Turkey [78], Hungary [79] and Serbia [62]. Contrary to other studies comparing organic and conventional wines [19], organic wines contained more Cd in average than their conventional counterparts (0.68 vs 0.28 ng/ml). The highest concentrations were found on El Hierro (1.27 ng/ml), almost 50% higher than all the other samples. White wines had three-fold higher Cd concentrations, attributable to different winemaking processes and greater use of clarifying and stabilising additives in whites [14].

#### Cr

Chromium in wines might result from contamination by stainless steel fermentation and storage tanks, or chromium oxides after bottling, rather than from soil accumulation [4]. The average 19.16 ng/ml is in line with previous studies [13, 73, 80], and much lower than results from other Spanish [81] and Greek contexts [69]. All samples were far from the maximum limit of 100 ng/ml established by the Croatian legislation, the highest concentration reached by GC2 being 43.85 ng/ml. Conventional wines presented slightly higher Cr levels than organic (23.79 vs 17.29 ng/ml). The eastern islands presented the highest Cr concentrations, led by Gran Canaria (30.20 ng/ml) and followed by Fuerteventura (28.37 ng/ml) and Lanzarote (21.26 ng/ml). No clear differences were found between red and white wines.

#### Fe

Iron can appear in wines as a result of presence in vineyard soils and environment, as well as from wine processing equipment [69]. It can also cause instability in wines at over 10,000 ng/ml, as shown in several studies [62, 79]. None of the samples exceeded the maximum OIV limits (10,000 ng/ml), with an overall average of 2,416.04 ng/ml. This is in line with averages reported in previous research [46, 60]. Organic wines had slightly higher Fe levels (2,602.30 vs 2,574.94 ng/ml), contradicting findings by Čepo et al. [19]. However, they are in agreement with Tobolková et al. [40], although differences were not significant in all cases. Instead, Dutra et al. [39] found no significant differences between Brazilian organic and conventional wines. The island with the highest Fe concentration was Gran Canaria, doubling Lanzarote’s average and six-fold higher than the lowest for Fuerteventura. The fact that both conventional and organic samples from Gran Canaria presented the highest Fe levels strongly suggests that geological and environmental origins are the key factors, above winemaking process contamination. Red wines presented significantly higher levels of Fe (3,574.17 vs 1,849.45 ng/ml).

#### Sb

The antimony content averaged 0.51 ng/ml, far below the OIV limit of 100 ng/ml. These levels are below most averages reported in the literature [46] but higher than levels in some South American wines [82]. Organic wines were higher in Sb (0.60 vs 0.49 ng/ml). The island with the greatest Sb levels was Gran Canaria, with a three-fold increased concentration over other islands. Red wines had higher Sb levels than whites (0.80 vs. 0.36).

#### Sn

The average value for tin of 3.85 ng/ml does not exceed any international limit by far and is below most reported studies on Sn levels, with averages of 15.20 ng/ml [15] and 17.83 ng/ml [82]. Averages regarding wine type, island and production method were distorted by the two conventional red wine samples (TF2 and GC2) having significantly higher Sn levels of 19.61 and 29.84, respectively. All other samples remained below the 1 ng/ml threshold.

#### Pb

Lead can be found in wine as a result of environmental pollution (industrial and exhaust fumes) and the corrosion of old metallic wine equipment during winemaking [83]. All samples were below the maximum limit of 150 ng/ml, with an average of 21.8 ng/ml. This is far from the highest levels reported in wines from Czech Republic [60], Germany [84], Hungary [79], and other Spanish locations [85]. Contrary to findings by Čepo et al. [19] and Alkış et al. [78], Pb was higher on average in organic than in conventional wines, almost doubling them. Instead, wine type was not a discriminant factor for Pb values, with white wines presenting slightly higher levels than red (25.62 vs 20.35 ng/ml). This opposes most data reported in the literature regarding lower Pb levels in whites, arguably because Pb is removed when grape skins and lead-attracting yeasts on them are discarded before fermentation, a procedure not commonly applied for reds [5].

#### Zn

Zinc is an essential element playing a central role in vine development, that appears naturally in wine [60]. Zn traces in wine can increase as a result of the use of pesticides and fertilisers containing it, especially superphosphate applications [86]. Excess Zn can have negative health effects and affect wine quality [15]. In our study, no sample exceeded the OIV limit of 5,000 ng/ml, with an average of 930.39 ng/ml. This is slightly higher than the averages found in wines globally, such as 640 ng/ml in Croatia [87] and 670 ng/ml in Kosovo [88]. Organic wines presented twice as much Zn as conventional ones (1,304.51 vs. 689.19 ng/ml). This is a striking difference not found in other comparative studies, which found no significant differences between the two production methods in Zn content [19]. White wine samples presented lower Zn contents than red (181.58 vs 1234.55 ng/ml), which can be explained by the use of bentonite for fining purposes, a process that lowers Zn concentrations [14]. Wines from Gran Canaria contained almost three times as much Zn (2,830.03 ng/ml) as the next highest island, Fuerteventura (1,146.57 ng/ml). The western islands of Tenerife, La Palma and El Hierro had the lowest concentrations.

## Conclusions

Few papers report comparisons between the elemental composition of conventional and organic wines. This is the first study to do so for the Canary Islands, although it reveals that the island factor is the only statistically significant variable to differentiate wine samples. Organic wines presented lower potential toxic element content on average than their conventional counterparts, but differences were not significant. On the contrary, there were more organic wines exceeding the maximum concentration limits established by the most restrictive international legislations. The results of this study thus confirm previous research showing no agreement between production method and wine’s elemental profile. Results are also in line with attempts reported in the literature to predict island of origin in the archipelago based on wine’s elemental composition. Comparisons with other bibliographical contexts have limited statistical meaning, given that there is little research examining the elemental composition of wines from volcanic geological zones and soils. More work is needed to shed light on their profiles, and on whether their differences derive from the soil or from specific winemaking techniques in the cellar. Further research should compare elemental profiles of natural and agricultural soils in the Canary Islands to better understand the origin of the presence of metals in the food chain, and wine in particular. Regarding the toxicological profile of the wines, five samples (three organic and two conventional) exceeded the maximum limits established in international legislation, highlighting the need for stricter analytical monitoring in the Canary Islands, with special focus on Cu and Ni. In many areas of the archipelago, agricultural soils are of volcanic origin, or with a high proportion of volcanic materials, such as serpentinites, which have a very high nickel content. Concentrations of REE and ME were consistently higher among conventional wines, but further research is needed to understand why and to confirm this tendency in other contexts.

## Supporting information

S1 Table Elemental analysis of organic (e.g TF1) vs conventional wine samples (e.g TF2) from each of the Canary Islands. Results are provided as means in ng/l followed by standard deviation.
(DOCX)

S2 Table Limits of Detection (LoDs) for each examined element. Results are provided as means in ng/l.
(XLSX)
