# Supplementary material for: Elemental composition, rare earths and minority elements in organic and conventional wines from volcanic areas: The Canary Islands (Spain)
Source: PLoS One. 2021 Nov 3;16(11):e0258739. doi: 10.1371/journal.pone.0258739 (PMC8565739; doi:10.1371/journal.pone.0258739)
Supplement: S1 Table — Results are provided as means in ng/l followed by standard deviation. (DOCX) [file pone.0258739.s001.docx]

| **Element** | **TF1** | **TF2** | **LP1** | **LP2** | **GC1** | **GC2** | **LG1** | **LG2** | **FT1** | **FT2** | **EH1** | **EH2** | **LZ1** | **LZ2** |
| --- | --- | --- | --- | --- | --- | --- | --- | --- | --- | --- | --- | --- | --- | --- |
| **56 Fe** | 1,990.02 ±  327.39 | 1,434.43 ±  117.02 | 2,610.54 ±  157.44 | 1,489.90 ±  101.35 | 6,912.25 ±  1765.70 | 4,904.02 ±  132.82 | 1,356.11 ±  380.22 | 2,462.80 ±  220.35 | 1,490.75 ±  149.34 | 1,087.23 ±  129.58 | 722.51 ±  169.02 | 3,575.75 ±  12.35 | 3,133.89  400.96 | 3,070.43 ±  345.95 |
| **63 Cu** | 34.04 ±  20.63 | 108.96 ±  50.95 | 328.18 ±  5.17 | 3.66 ±  0.64 | 93.31 ±  4.54 | 331.01 ±  79.72 | 163.79 ±  107.98 | 135.46 ±  18.43 | 600.59 ±  282.86 | 1,228.25 ±  62.38 | 97.65 ±  48.36 | 19.14 ±  8.72 | 20.39 ±  16.79 | 24.40 ±  4.10 |
| **66 Zn** | 706.80 ±  25.43 | 242.66 ±  8.50 | 866.11 ±  151.16 | 126.06 ±  5.09 | 3,973.52 ±  77.72 | 1,686.53 ±  13.69 | 947.77 ±  166.78 | 1,223.34 ±  12.75 | 1,865.87 ±  322.54 | 427.27 ±  20.36 | 326.77 ±  32.78 | 765.41 ±  24.92 | 444.70 ±  19.80 | 353.06 ±  8.31 |
| **78 Se** | 0.87 ±  0.69 | 0.26 ±  0.11 | 1.17 ±  0.34 | 1.24 ±  0.79 | 1.56 ±  0.34 | 4.72 ±  1.57 | 1.41 ±  0.68 | 0.97 ±  1.69 | 0.00 ±  0.00 | 0.21 ±  0.37 | 0.00 ±  0.00 | 0.00 ±  0.00 | 0.81 ±  1.21 | 1.74 ±  0.48 |
| **55 Mn** | 782.45 ±  18.88 | 1,955.88 ±  11.15 | 372.98 ±  10.52 | 722.53 ±  1.45 | 1,928.99 ±  40.79 | 1,377.31 ±  12.46 | 1,073.67 ±  39.54 | 2,211.77 ±  22.44 | 519.97 ±  18.48 | 758.36 ±  27.94 | 678.23 ±  15.87 | 1,511.97 ±  9.43 | 583.93 ±  23.27 | 622.01 ±  10.51 |
| **75 As** | 0.47 ±  0.11 | 0.34 ±  0.03 | 0.82 ±  0.05 | 1.54 ±  0.29 | 0.79 ±  0.04 | 4.03 ±  0.14 | 0.27 ±  0.13 | 1.12 ±  0.27 | 2.35 ±  0.70 | 1.22 ±  0.21 | 1.37 ±  0.08 | 0.28 ±  0.03 | 3.51 ±  0.38 | 4.28 ±  0.19 |
| **111 Cd** | 0.22 ±  0.03 | 0.34 ±  0.06 | 1.00 ±  0.45 | 0.15 ±  0.01 | 0.22 ±  0.02 | 0.33 ±  0.03 | 0.31 ±  0.10 | 0.37 ±  0.10 | 0.52 ±  0.12 | 0.30 ±  0.01 | 2.32 ±  1.69 | 0.22 ±  0.01 | 0.19 ±  0.03 | 0.23 ±  0.01 |
| **202 Hg** | 0.14 ±  0.08 | 0.13 ±  0.06 | 0.29 ±  0.26 | 0.05 ±  0.07 | 0.05 ±  0.08 | 0.00 ±  0.00 | 0.10 ±  0.16 | 0.29 ±  0.10 | 0.01 ±  0.00 | 0.01 ±  0.00 | 0.01 ±  0.00 | 0.01 ±  0.00 | 0.01 ±  0.00 | 0.01 ±  0.00 |
| **208 Pb** | 12.98 ±  0.33 | 3.21 ±  0.52 | 11.71 ±  2.92 | 3.41 ±  0.15 | 69.06 ±  3.23 | 23.14 ±  2.28 | 43.25 ±  24.78 | 52.11 ±  2.81 | 78.19 ±  1.23 | 2.70 ±  0.31 | 7.26 ±  0.85 | 6.33 ±  1.05 | 8.48 ±  0.31 | 5.26 ±  0.43 |
| **107 Ag** | 0.06 ±  0.02 | 0.09 ±  0.01 | 0.18 ±  0.08 | 0.31 ±  0.35 | 0.04 ±  0.07 | 0.16 ±  0.04 | 0.06 ±  0.06 | 0.04 ±  0.06 | 0.04 ±  0.06 | 0.12 ±  0.02 | 0.04 ±  0.06 | 0.00 ±  0.00 | 0.07 ±  0.12 | 0.08 ±  0.07 |
| **27 Al** | 1.055.89 ±  275.76 | 673.42 ±  162.40 | 753.26 ±  170.81 | 966.65 ±  27.12 | 1,632.68 ±  191.96 | 2,255.14 ±  58.34 | 928.52 ±  386.48 | 1,051.63 ±  400.84 | 1,229.07 ±  248.59 | 1,159.12 ±  167.97 | 1,027.11 ±  269.67 | 786.42 ±  108.83 | 1,952.61 ±  76.30 | 1,798.24 ±  274.99 |
| **197 Au** | 0.17 ±  0.05 | 0.04 ±  0.06 | 0.00 ±  0.00 | 0.00 ±  0.00 | 0.00 ±  0.00 | 0.00 ±  0.00 | 0.00 ±  0.00 | 0.32 ±  0.03 | 0.01 ±  0.00 | 0.01 ±  0.00 | 0.01 ±  0.00 | 0.01 ±  0.00 | 0.01 ±  0.00 | 0.01 ±  0.00 |
| **137 Ba** | 98.26 ±  3.93 | 135.13 ±  6.81 | 144.29 ±  19.83 | 161.85 ±  2.29 | 81.26 ±  21.03 | 328.41 ±  92.25 | 227.05 ±  17.33 | 153.01 ±  3.11 | 124.37 ±  11.50 | 106.09 ±  5.24 | 82.77 ±  5.83 | 70.03 ±  3.64 | 131.50 ±  10.66 | 128.30 ±  8.80 |
| **9 Be** | 0.23 ±  0.06 | 0.15 ±  0.10 | 0.28 ±  0.07 | 0.55 ±  0.02 | 0.86 ±  0.07 | 0.56 ±  0.07 | 0.17 ±  0.10 | 0.64 ±  0.11 | 0.11 ±  0.19 | 0.31 ±  0.04 | 0.10 ±  0.09 | 0.04 ±  0.06 | 0.10 ±  0.16 | 0.12 ±  0.00 |
| **59 Co** | 1.22 ±  0.09 | 2.29 ±  0.11 | 5.00 ±  0.21 | 3.69 ±  0.02 | 4.02 ±  0.07 | 22.11 ±  0.13 | 8.78 ±  0.34 | 4.38 ±  0.33 | 7.75 ±  0.16 | 6.88 ±  0.30 | 2.45 ±  0.19 | 3.63 ±  0.15 | 5.10 ±  0.38 | 3.92 ±  0.13 |
| **52 Cr** | 13.37 ±  1.04 | 10.63 ±  0.55 | 13.59 ±  2.80 | 9.35 ±  0.58 | 16.55 ±  0.99 | 43.85 ±  1.53 | 12.12 ±  0.69 | 27.67 ±  21.64 | 24.40 ±  2.66 | 32.25 ±  1.59 | 17.20 ±  0.51 | 24.03 ±  0.62 | 23.77 ±  1.23 | 18.76 ±  0.61 |
| **95 Mo** | 1.68 ±  0.30 | 0.96 ±  0.04 | 1.12 ±  0.13 | 0.08 ±  0.11 | 2.47 ±  0.20 | 10.33 ±  0.14 | 1.63 ±  0.30 | 0.87 ±  0.08 | 7.34 ±  0.78 | 3.58 ±  0.12 | 1.88 ±  0.07 | 8.08 ±  0.06 | 8.26 ±  0.54 | 3.90 ±  0.29 |
| **60 Ni** | 16.68 ±  1.57 | 22.50 ±  16.61 | 17.28 ±  1.20 | 4.07 ±  6.51 | 419.63 ±  2.29 | 57.73 ±  0.74 | 113.04 ±  14.81 | 99.05 ±  120.75 | 101.95 ±  20.42 | 1.81 ±  0.00 | 1.81 ±  0.00 | 161.13 ±  1.31 | 1.81 ±  0.00 | 1.81 ±  0.00 |
| **121 Sb** | 0.31 ±  0.30 | 0.14 ±  0.14 | 0.75 ±  0.32 | 0.24 ±  0.09 | 1.20 ±  0.46 | 1.92 ±  1.24 | 0.32 ±  0.06 | 0.59 ±  1.00 | 0.75 ±  0.09 | 0.18 ±  0.28 | 0.02 ±  0.00 | 0.02 ±  0.00 | 0.87 ±  0.02 | 0.34 ±  0.28 |
| **118 Sn** | 0.76 ±  0.00 | 19.61 ±  32.64 | 0.76 ±  0.00 | 0.76 ±  0.00 | 0.76 ±  0.00 | 29.84 ±  6.39 | 0.76 ±  0.00 | 0.65 ±  0.00 | 0.65 ±  0.00 | 0.65 ±  0.00 | 0.65 ±  0.00 | 0.65 ±  0.00 | 0.65 ±  0.00 | 0.65 ±  0.00 |
| **88 Sr** | 736.70 ±  13.28 | 841.74 ±  7.91 | 1,777.17 ±  24.71 | 1,572.63 ±  21.08 | 1,426.38 ±  89.80 | 1,257.06 ±  85.92 | 611.74 ±  51.34 | 769.05 ±  113.69 | 409.36 ±  9.41 | 792.62 ±  26.73 | 826.34 ±  10.63 | 1,125.21 ±  6.88 | 758.21 ±  28.86 | 674.69 ±  8.62 |
| **232 Th** | 0.04 ±  0.06 | 0.00 ±  0.00 | 0.08 ±  0.07 | 0.19 ±  0.06 | 0.06 ±  0.06 | 0.19 ±  0.07 | 0.16 ±  0.19 | 0.21 ±  0.36 | 0.13 ±  0.08 | 0.22 ±  0.02 | 0.25 ±  0.03 | 0.03 ±  0.04 | 0.16 ±  0.26 | 0.16 ±  0.05 |
| **47 Ti** | 27.08 ±  22.91 | 40.98 ±  35.53 | 42.32 ±  38.83 | 77.66 ±  15.62 | 39.33 ±  33.87 | 132.88 ±  31.42 | 71.77 ±  62.27 | 36.45 ±  61.29 | 53.74 ±  61.68 | 84.22 ±  21.69 | 92.82 ±  23.21 | 40.90 ±  36.16 | 45.77 ±  13.01 | 96.34 ±  36.44 |
| **205 Tl** | 0.23 ±  0.02 | 0.29 ±  0.01 | 0.22 ±  0.00 | 0.32 ±  0.01 | 0.08 ±  0.01 | 0.12 ±  0.01 | 0.53 ±  0.03 | 0.94 ±  0.03 | 0.34 ±  0.05 | 0.16 ±  0.13 | 0.24 ±  0.02 | 0.60 ±  0.03 | 0.24 ±  0.02 | 0.22 ±  0.01 |
| **238 U** | 0.03 ±  0.03 | 0.06 ±  0.04 | 0.05 ±  0.02 | 0.17 ±  0.03 | 0.12 ±  0.01 | 0.29 ±  0.02 | 0.04 ±  0.03 | 0.50 ±  0.02 | 0.06 ±  0.04 | 0.27 ±  0.01 | 0.59 ±  0.02 | 0.16 ±  0.12 | 0.15 ±  0.03 | 0.14 ±  0.03 |
| **51 V** | 1.02 ±  0.32 | 1.67 ±  0.23 | 7.51 ±  0.70 | 4.95 ±  0.55 | 2.33 ±  0.57 | 16.36 ±  0.30 | 3.80 ±  0.90 | 4.46 ±  1.31 | 6.84 ±  0.95 | 8.63 ±  0.83 | 20.82 ±  0.85 | 7.67 ±  0.83 | 35.06 ±  1.74 | 17.89 ±  0.70 |
| **71 Ga** | 0.22 ±  0.09 | 0.21 ±  0.07 | 0.36 ±  0.16 | 0.81 ±  0.66 | 0.52 ±  0.16 | 1.82 ±  0.21 | 0.36 ±  0.30 | 0.51 ±  0.47 | 0.36 ±  0.18 | 0.45 ±  0.06 | 0.41 ±  0.08 | 0.31 ±  0.08 | 0.63 ±  0.20 | 0.67 ±  0.17 |
| **89 Y** | 0.23 ±  0.10 | 0.30 ±  0.09 | 1.63 ±  1.59 | 1.31 ±  0.46 | 2.10 ±  2.57 | 14.67 ±  18.41 | 1.46 ±  2.01 | 6.84 ±  10.48 | 0.57 ±  0.37 | 1.33 ±  0.17 | 1.64 ±  0.15 | 0.48 ±  0.21 | 2.52 ±  3.24 | 1.32 ±  0.46 |
| **93 Nb** | 1.65 ±  1.13 | 1.27 ±  0.07 | 0.92 ±  1.13 | 2.42 ±  1.32 | 2.97 ±  0.90 | 4.01 ±  0.60 | 2.04 ±  2.14 | 2.35 ±  1.15 | 1.73 ±  1.43 | 2.48 ±  0.58 | 2.27 ±  0.94 | 0.88 ±  0.74 | 1.90 ±  0.47 | 2.67 ±  1.13 |
| **101 Ru** | 0.00 ±  0.00 | 0.00 ±  0.00 | 0.00 ±  0.00 | 0.00 ±  0.00 | 0.00 ±  0.00 | 0.00 ±  0.00 | 0.00 ±  0.00 | 0.00 ±  0.00 | 0.00 ±  0.00 | 0.00 ±  0.00 | 0.00 ±  0.00 | 0.00 ±  0.00 | 0.00 ±  0.00 | 0.00 ±  0.00 |
| **115 In** | 0.00 ±  0.00 | 0.00 ±  0.15 | 0.00 ±  0.00 | 0.00 ±  0.00 | 0.06 ±  0.09 | 0.08 ±  0.08 | 0.00 ±  0.00 | 0.00 ±  0.00 | 0.07 ±  0.03 | 0.00 ±  0.00 | 0.00 ±  0.00 | 0.00 ±  0.00 | 0.00 ±  0.00 | 0.00 ±  0.00 |
| **139 La** | 0.26 ±  0.19 | 0.40 ±  0.09 | 0.97 ±  1.05 | 4.01 ±  4.62 | 0.80 ±  0.25 | 3.02 ±  3.73 | 0.90 ±  0.92 | 2.51 ±  3.83 | 0.76 ±  0.76 | 2.01 ±  0.52 | 2.23 ±  0.38 | 0.73 ±  0.45 | 2.72 ±  3.44 | 1.65 ±  0.88 |
| **140 Ce** | 0.69 ±  0.47 | 1.16 ±  0.51 | 2.15 ±  1.85 | 8.71 ±  10.93 | 2.63 ±  1.69 | 4.90 ±  3.17 | 2.34 ±  2.64 | 3.28 ±  4.44 | 1.50 ±  1.47 | 3.60 ±  0.45 | 2.99 ±  0.95 | 1.21 ±  0.70 | 2.53 ±  1.13 | 3.71 ±  1.90 |
| **141 Pr** | 0.05 ±  0.04 | 0.10 ±  0.03 | 0.30 ±  0.34 | 1.11 ±  1.42 | 0.22 ±  0.12 | 0.77 ±  0.77 | 0.24 ±  0.27 | 0.67 ±  1.00 | 0.20 ±  0.21 | 0.49 ±  0.10 | 0.52 ±  0.08 | 0.19 ±  0.13 | 0.69 ±  0.88 | 0.44 ±  0.22 |
| **146 Nd** | 0.21 ±  0.16 | 0.39 ±  0.15 | 1.35 ±  1.52 | 4.75 ±  6.30 | 0.96 ±  0.63 | 3.62 ±  2.91 | 1.00 ±  1.16 | 2.86 ±  4.32 | 0.74 ±  0.83 | 1.91 ±  0.35 | 1.99 ±  0.33 | 0.74 ±  0.51 | 2.78 ±  3.55 | 1.70 ±  0.81 |
| **147 Sm** | 0.03 ±  0.04 | 0.08 ±  0.03 | 0.27 ±  0.32 | 0.89 ±  1.16 | 0.20 ±  0.14 | 0.94 ±  0.58 | 0.20 ±  0.23 | 0.63 ±  0.94 | 0.14 ±  0.16 | 0.36 ±  0.06 | 0.39 ±  0.05 | 0.15 ±  0.11 | 0.51 ±  0.64 | 0.35 ±  0.18 |
| **153 Eu** | 0.01 ±  0.01 | 0.02 ±  0.01 | 0.09 ±  0.10 | 0.23 ±  0.28 | 0.06 ±  0.06 | 0.35 ±  0.25 | 0.07 ±  0.07 | 0.22 ±  0.35 | 0.04 ±  0.05 | 0.11 ±  0.02 | 0.09 ±  0.02 | 0.04 ±  0.03 | 0.14 ±  0.19 | 0.11 ±  0.05 |
| **157 Gd** | 0.03 ±  0.02 | 0.06 ±  0.02 | 0.26 ±  0.30 | 0.61 ±  0.70 | 0.21 ±  0.18 | 1.30 ±  1.07 | 0.20 ±  0.23 | 0.80 ±  1.20 | 0.12 ±  0.13 | 0.32 ±  0.06 | 0.36 ±  0.04 | 0.13 ±  0.09 | 0.49 ±  0.62 | 0.31 ±  0.14 |
| **159 Tb** | 0.00 ±  0.00 | 0.01 ±  0.00 | 0.04 ±  0.04 | 0.07 ±  0.06 | 0.03 ±  0.03 | 0.21 ±  0.19 | 0.03 ±  0.04 | 0.19 ±  0.19 | 0.02 ±  0.02 | 0.04 ±  0.01 | 0.05 ±  0.01 | 0.02 ±  0.01 | 0.07 ±  0.09 | 0.05 ±  0.02 |
| **163 Dy** | 0.03 ±  0.02 | 0.04 ±  0.02 | 0.23 ±  0.24 | 0.27 ±  0.17 | 0.24 ±  0.26 | 1.53 ±  1.55 | 0.20 ±  0.25 | 0.83 ±  1.24 | 0.10 ±  0.08 | 0.24 ±  0.03 | 0.29 ±  0.04 | 0.09 ±  0.06 | 0.41 ±  0.50 | 0.27 ±  0.10 |
| **165 Ho** | 0.00 ±  0.01 | 0.01 ±  0.00 | 0.05 ±  0.05 | 0.05 ±  0.02 | 0.06 ±  0.06 | 0.39 ±  0.44 | 0.04 ±  0.06 | 0.18 ±  0.27 | 0.02 ±  0.01 | 0.05 ±  0.01 | 0.06 ±  0.01 | 0.02 ±  0.01 | 0.08 ±  0.10 | 0.05 ±  0.02 |
| **166 Er** | 0.02 ±  0.01 | 0.03 ±  0.01 | 0.12 ±  0.11 | 0.11 ±  0.03 | 0.17 ±  0.19 | 1.15 ±  1.33 | 0.12 ±  0.16 | 0.52 ±  0.76 | 0.05 ±  0.04 | 0.13 ±  0.02 | 0.16 ±  0.02 | 0.04 ±  0.02 | 0.22 ±  0.25 | 0.13 ±  0.04 |
| **169 Tm** | 0.00 ±  0.00 | 0.00 ±  0.00 | 0.02 ±  0.01 | 0.02 ±  0.00 | 0.03 ±  0.02 | 0.16 ±  0.17 | 0.02 ±  0.02 | 0.07 ±  0.09 | 0.01 ±  0.01 | 0.02 ±  0.00 | 0.02 ±  0.00 | 0.01 ±  0.00 | 0.03 ±  0.03 | 0.02 ±  0.00 |
| **172 Yb** | 0.02 ±  0.01 | 0.02 ±  0.01 | 0.09 ±  0.07 | 0.11 ±  0.02 | 0.14 ±  0.12 | 0.84 ±  0.80 | 0.09 ±  0.12 | 0.37 ±  0.48 | 0.05 ±  0.03 | 0.10 ±  0.01 | 0.14 ±  0.02 | 0.03 ±  0.01 | 0.18 ±  0.18 | 0.10 ±  0.03 |
| **175 Lu** | 0.00 ±  0.00 | 0.00 ±  0.00 | 0.02 ±  0.01 | 0.02 ±  0.00 | 0.02 ±  0.02 | 0.14 ±  0.12 | 0.01 ±  0.02 | 0.06 ±  0.08 | 0.01 ±  0.00 | 0.02 ±  0.00 | 0.03 ±  0.00 | 0.01 ±  0.00 | 0.03 ±  0.03 | 0.02 ±  0.00 |
| **181 Ta** | 0.54 ±  0.89 | 0.03 ±  0.00 | 0.03 ±  0.00 | 0.03 ±  0.00 | 0.03 ±  0.00 | 0.03 ±  0.00 | 0.03 ±  0.00 | 2.02 ±  0.22 | 0.87 ±  0.14 | 1.02 ±  0.34 | 0.80 ±  0.14 | 0.02 ±  0.00 | 1.67 ±  0.94 | 0.49 ±  0.41 |
| **189 Os** | 0.01 ±  0.01 | 0.00 ±  0.00 | 0.00 ±  0.00 | 0.00 ±  0.00 | 0.00 ±  0.00 | 0.00 ±  0.00 | 0.00 ±  0.00 | 0.00 ±  0.00 | 0.00 ±  0.00 | 0.00 ±  0.00 | 0.00 ±  0.00 | 0.00 ±  0.00 | 0.00 ±  0.00 | 0.00 ±  0.00 |
| **195 Pt** | 0.09 ±  0.09 | 0.00 ±  0.00 | 0.00 ±  0.00 | 0.00 ±  0.00 | 0.00 ±  0.00 | 0.00 ±  0.00 | 0.00 ±  0.00 | 0.03 ±  0.04 | 0.00 ±  0.00 | 0.00 ±  0.00 | 0.00 ±  0.00 | 0.00 ±  0.00 | 0.00 ±  0.00 | 0.00 ±  0.00 |
| **209 Bi** | 0.00 ±  0.00 | 0.05 ±  0.04 | 0.17 ±  0.09 | 0.11 ±  0.09 | 0.00 ±  0.00 | 0.11 ±  0.03 | 0.10 ±  0.09 | 0.06 ±  0.01 | 0.14 ±  0.02 | 0.03 ±  0.02 | 0.11 ±  0.04 | 0.00 ±  0.00 | 0.09 ±  0.04 | 0.05 ±  0.00 |
